# Ethanolic Extract of *Senna velutina* Roots: Chemical Composition, *In Vitro* and *In Vivo* Antitumor Effects, and B16F10-Nex2 Melanoma Cell Death Mechanisms

**DOI:** 10.1155/2019/5719483

**Published:** 2019-06-12

**Authors:** David Tsuyoshi Hiramatsu Castro, Jaqueline Ferreira Campos, Marcio José Damião, Heron Fernandes Vieira Torquato, Edgar Julian Paredes-Gamero, Carlos Alexandre Carollo, Elaine Guadelupe Rodrigues, Kely de Picoli Souza, Edson Lucas dos Santos

**Affiliations:** ^1^Research Group on Biotechnology and Bioprospecting Applied to Metabolism (GEBBAM), Federal University of Grande Dourados, Dourados, CEP: 79804-970 MS, Brazil; ^2^Department of Biochemistry, Federal University of São Paulo, São Paulo, CEP: 04044-020, SP, Brazil; ^3^Faculty of Pharmaceutical Sciences, Food and Nutrition, Federal University of Mato Grosso do Sul, Campo Grande, CEP: 79070-900, MS, Brazil; ^4^Laboratory of Natural Products and Mass Spectrometry, Federal University of Mato Grosso do Sul, Campo Grande, CEP: 79070-900 MS, Brazil; ^5^Department of Microbiology, Immunology, and Parasitology, Paulista School of Medicine, Federal University of São Paulo (EPM-UNIFESP), São Paulo, CEP: 04023-062 SP, Brazil

## Abstract

Cutaneous melanoma is among the most aggressive types of cancer, and its rate of occurrence increases every year. Current pharmacological treatments for melanoma are not completely effective, requiring the identification of new drugs. As an alternative, plant-derived natural compounds are described as promising sources of new anticancer drugs. In this context, the objectives of this study were to identify the chemical composition of the ethanolic extract of *Senna velutina* roots (ESVR), to assess its *in vitro* and *in vivo* antitumor effects on melanoma cells, and to characterize its mechanisms of action. For these purposes, the chemical constituents were identified by liquid chromatography coupled to high-resolution mass spectrometry. The *in vitro* activity of the extract was assessed in the B16F10-Nex2 melanoma cell line using the 3-(4,5-dimethylthiazol-2-yl)-2,5-diphenyltetrazolium bromide (MTT) assay and based on the apoptotic cell count; DNA fragmentation; necrostatin-1 inhibition; intracellular calcium, pan-caspase, and caspase-3 activation; reactive oxygen species (ROS) levels; and cell cycle arrest. The *in vivo* activity of the extract was assessed in models of tumor volume progression and pulmonary nodule formation in C57Bl/6 mice. The chemical composition results showed that ESVR contains flavonoid derivatives of the catechin, anthraquinone, and piceatannol groups. The extract reduced B16F10-Nex2 cell viability and promoted apoptotic cell death as well as caspase-3 activation, with increased intracellular calcium and ROS levels as well as cell cycle arrest at the sub-G_0_/G_1_ phase. *In vivo*, the tumor volume progression and pulmonary metastasis of ESVR-treated mice decreased over 50%. Combined, these results show that ESVR had *in vitro* and *in vivo* antitumor effects, predominantly by apoptosis, thus demonstrating its potential as a therapeutic agent in the treatment of melanoma and other types of cancer.

## 1. Introduction

Cancer is among the leading causes of death worldwide [1]. In particular, cutaneous melanoma is a potentially lethal form of skin cancer and occurs when melanocytes, cells responsible for producing the melanin pigment, undergo changes mediated by endogenous and/or exogenous events, thereby becoming malignant [2, 3]. The main factors responsible for the onset of melanoma are intrinsic and extrinsic. Intrinsic factors primarily include genetic susceptibility and family history, whereas the main extrinsic factor is excessive exposure to ultraviolet radiation [4, 5].

In recent decades, the incidence of cutaneous melanoma has increased, and according to the World Health Organization, approximately 132,000 cases of melanoma are diagnosed every year worldwide [6]. Its incidence varies among different populations, and the highest rates are reported in countries such as Australia and New Zealand [7]. When melanoma is detected early, surgical removal increases the treatment efficacy in approximately 99% of cases [8]. Chemotherapy, immunotherapy, and molecular therapy are among the main treatments for melanoma [9, 10]. Although patient survival rates are increasing, therapies and their combinations are still limited because they cause toxicity [11]. In addition, advanced-stage melanoma is resistant to drug therapy [12].

As an alternative to current therapies, phytochemical molecules have gained prominence as promising agents for the development of new drugs in the treatment of neoplasia [13]. Some studies have demonstrated that these substances show low toxicity in normal cells and act as melanoma treatment adjuvants, enhancing the anticancer effects of chemotherapeutic agents [14, 15].

In the scientific literature, the anticancer properties of more than 3000 plant species have been described [16]. Furthermore, in the last 70 years, 175 anticancer molecules were approved by the Food and Drug Administration (FDA), and 85 of them are derived from natural products or their derivatives [17].

These molecules, known as secondary metabolites, are complex compounds with diverse structures responsible for various biological activities [18]. These characteristics, together with the high degree of biodiversity in Brazil, may provide a promising source of new drugs. The genus *Senna* (Fabaceae) is found in the Brazilian Cerrado and has more than 250 species whose antimicrobial [19], antidiabetic [20], antioxidant [21], anti-inflammatory [22], and anticancer [23–25] properties have been described.

The species *Senna velutina*, a shrub of the genus *Senna*, commonly known as São João, vermelhinho, or Fedegoso-do-Cerrado, is found in the Central-West, Southeast, and Northeast regions of Brazil [26, 27]. Although the species is used by the population for medicinal purposes, only one article has described the antileukemic activity of the leaves of this plant [24]. However, no scientific study has described the chemical constituents and anticancer properties of the roots of this plant. In this context, the objective of this study was to characterize the chemical composition, assess the *in vitro* and *in vivo* antitumor effects, and identify the mechanisms through which the ethanolic extract of *S. velutina* roots (ESVR) promotes B16F10-Nex2 melanoma cell death.

## 2. Materials and Methods

### 2.1. Plant Material and Extract Preparation

*S. velutina*roots were collected in the Cerrado region (Brazilian biome), in the state of Mato Grosso do Sul (S 22° 05′ 545^″^ and W 055° 20′ 746^″^), in the Central-West region of Brazil and identified by a botanist. A dried sample of the species was deposited in the Herbarium of the Federal University of Grande Dourados-UFGD, Dourados, Mato Grosso do Sul (MS), Brazil, with registration number 4665. Root collection was authorized by the Sistema de Autorização e Informação em Biodiversidade (Biodiversity Information and Authorization System; SISBIO, permit number 54470-1). Subsequently, the plant roots were rinsed, dried in an air circulation oven for 15 days at 36°C, and pulverized; 200 g was then macerated in 95% ethanol (7: 1) at room temperature for 7 days. Then, the extract was filtered and the residue was subjected to the same procedure twice. After 21 days, the filtrate was concentrated in a rotary vacuum evaporator (Gehaka, São Paulo, SP, Brazil) and subsequently freeze-dried (model Savant MicroModulyo, Thermo Fisher Scientific, Massachusetts, EUA). The dry extract yield was 23%, calculated using the following formula: extraction yield (%) = (weight of the freeze − dried extract × 100)/(weight of the original sample). The ESVR was stored at -20°C for subsequent experiments.

### 2.2. Phytochemical Analysis

ESVR was analyzed in an ultrafast liquid chromatograph (UFLC, Shimadzu) coupled to a diode array detector (DAD, Shimadzu) and electrospray ionization time-of-flight mass spectrometer (ESI-QTOF-micrOTOF QII, Bruker Daltonics; operating in the positive and negative ionization modes, 120-1200 Da). A C-18 column was used (Kinetex, 2.6 *μ*m, 150 × 2.2 mm, Phenomenex), protected by a guard precolumn of the same material. The mobile phase was water (solvent A) and acetonitrile (solvent B), both with 0.1% formic acid, in a gradient of 0-2 min 3% B, 2-25 min 3-25% B, and 25-40 min 25-80% B, followed by the washing and reconditioning of the columns (8 min). The flow rate was 0.3 mL/min, and 1 *μ*L of the extract (1 mg/mL) was injected. The other micrOTOF QII parameters were as follows: temperature, 200°C; N_2_ gas flow rate, 9 L/min; nebulizer, 4.0 bar; capillary voltage, 3500 V (negative), +4500 V (positive); and internal calibration with sodium trifluoroacetate (TFA-Na) injected at the end of the chromatographic analysis. The catechin and piceatannol authentic standards were purchased from Sigma-Aldrich with ≥95% purity. The metabolites present in ESVR were identified based on the interpretation of mass and UV absorption spectra and based on comparison with the literature. When available, the compounds were confirmed by comparison with authentic standards.

### 2.3. Cell Lines and Cell Cultures

Human peripheral blood mononuclear cells (PBMCs) were collected after donor consent. Mononuclear cells were separated by centrifugation using Ficoll Histopaque-1077 (1.077 g/cm^3^) (Sigma-Aldrich, Germany) according to the manufacturer's instructions at 400 × g for 30 min. The use of human blood was approved by the Ethics Committee of the Federal University of Grande Dourados (UFGD) under protocol 123/12. The murine melanoma subline (B16F10-Nex2) was isolated at the Oncology Experimental Unit (Federal University of São Paulo, UNIFESP) from the B16F10 cell line and cultured in RPMI 1640 medium (Gibco/Invitrogen, Minneapolis, MN) supplemented with 4-(2-hydroxyethyl)piperazine-1-ethanesulfonic acid (HEPES, 10 nM) and sodium bicarbonate (24 nM). Human lung fibroblasts (MRC-5) and human melanoma cell lines (SK-Mel-28 and SK-Mel-103) were cultured in high-glucose Dulbecco's Modified Eagle's Medium (DMEM). All cell lines were supplemented with 10% fetal bovine serum (FBS, purchased from Gibco/Invitrogen) and 40 mg/mL gentamicin (Hipolabor Farmacêutica, Sabará, MG, Brazil). They were kept in flasks at 37°C in 5% CO_2_.

### 2.4. MTT Cell Viability Assay

The cell viability was assessed using the 3-(4,5-diphenyltetrazolium-2-yl)-2,5-diphenyltrazolium bromide (MTT) colorimetric assay. Adherent cells were plated at a density of 5 × 10^3^ cells/well, and PBMCs were plated at 10^5^ cells/well in 96-well microtiter plates. After 24 and 48 h, solutions with different ESVR concentrations (25-125 *μ*g/mL), diluted in medium with 0.1% DMSO, were added, and medium with only 0.1% DMSO was used as a control. At the end of both periods, 100 *μ*L MTT (0.5 mg/mL) was added to each well. The cell culture was incubated for another 4 h, and 100 *μ*L of DMSO was then added to solubilize the formazan crystals. The absorbance was determined at 570 nm using a SpectraMax 250 reader (Molecular Devices). Cell viability inhibition was calculated using the following formula:

(1) $$\begin{array}{c} \text{Cell viability }\left(\%\right)=\left[{\text{Abs}}_{\text{treated cells}}/{\text{Abs}}_{\text{control}}\right]\times 100. \end{array}$$

### 2.5. Effect of ESVR on B16F10-Nex2 Cells

B16F10-Nex2 cells (5 × 10^3^ cells/well) were subjected to solutions with different ESVR concentrations (25-125 *μ*g/mL) diluted in RPMI 1640 solution with 0.1% DMSO for 24 h. RPMI 1640 solution with 0.1% DMSO was used as a control. Subsequently, cell images were acquired under a Nikon TE2000E (Nikon Instruments Inc.) microscope (10x objective).

### 2.6. Cell Death Profile

The cell death profile was determined using the method described by Paredes-Gamero et al. in 2012 [28] with a few modifications. B16F10-Nex2 cells were plated in 48-well plates (10^4^ cells/well) and cultured in RPMI 1640 with 10% FBS for 24 h as well as with the half-maximal inhibitory concentration (IC_50_) of ESVR (52 *μ*g/mL). After this period, the cells were washed with phosphate-buffered saline (PBS), detached, and resuspended in buffer solution (0.01 M HEPES, pH = 7.4, 0.14 M NaCl, and 2.5 mM CaCl_2_). The suspension was labeled with annexin V-fluorescein isothiocyanate (FITC) and propidium iodide (Becton Dickinson, USA) according to the manufacturer's instructions. The cells were incubated with the IC_50_ concentration of ESVR diluted in the medium to assess whether the extract showed fluorescence under the study parameters. The cells were incubated for 15 min at room temperature, and subsequently, 10,000 events per sample were collected and analyzed in the Accuri™ C6 flow cytometer (Becton Dickinson, USA) using the software FlowJo v10.2 LCC (Oregon, USA).

### 2.7. Apoptotic B16F10-Nex2 Cell Nuclei Count

To count the apoptotic cell nuclei, 6 × 10^4^ B16F10-Nex2 cells/well were plated on coverslips in 24-well plates. Subsequently, the cells were treated with 52 *μ*g/mL ESVR diluted in RPMI 1640 solution with 0.1% DMSO for 24 h. As a control, RPMI 1640 solution with 0.1% DMSO was used. After this period, the supernatant was discarded, and the cells were washed with PBS twice and fixed with 2% paraformaldehyde for 20 min at room temperature. Then, the cells were washed with PBS and permeabilized with 0.01% saponin for 20 min.

To detect apoptotic nuclei, coverslips were placed on slides and labeled with 4′-6′-diamidino-2-phenylindole (DAPI) dihydrochloride. Cells were counted under a LEICA DMI 6000B confocal microscope (Leica Microsystems, Germany).

### 2.8. DNA Fragmentation

B16F10-Nex2 cells were plated at 1 × 10^6^ cells/well. After 24 h, the cells were treated with 52 *μ*g/mL ESVR diluted in RPMI 1640 solution with 0.1% DMSO. RPMI 1640 solution with 0.1% DMSO was used as a control for 12 and 24 h. Subsequently, the DNA was extracted with phenol/chloroform, followed by incubation with RNAse (20 *μ*g/mL). The DNA integrity was visualized in a 2.5% agarose gel stained with ethidium bromide (0.5 *μ*g/mL).

### 2.9. Caspase-3 Activity

Caspase-3 activation was assessed by flow cytometry according to the method described by Moraes et al. in 2013 [29] with minor modifications. B16F10-Nex2 cells (6 × 10^4^ cells/well) were treated with ESVR (52 *μ*g/mL) and diluted in RPMI 1640 solution with 0.1% DMSO for 24 h. RPMI 1640 solution with 0.1% DMSO was used as a control. After the treatment, the cells were fixed with 2% paraformaldehyde for 30 min and then permeabilized with 0.01% saponin in PBS for 20 min at room temperature. Subsequently, the cells were incubated for 1 h with cleaved caspase-3 (Asp175) antibody (Alexa Fluor® 488 conjugate) at room temperature and protected from light. After the incubation period, the fluorescence was acquired in 10,000 events in the Accuri C6 flow cytometer (Becton Dickinson, San Jose, CA) and analyzed using the software FlowJo v8.7 (Ashland, USA).

### 2.10. Determination of the Reactive Oxygen Species (ROS) Levels

ROS levels were determined by flow cytometry using the 2′,7′-dichlorodihydrofluorescein diacetate (H_2_DCFDA) dye (Molecular Probe-Life Technologies, Carlsbad, CA). For this purpose, B16F10-Nex2 cells (6 × 10^4^ cells/well) were treated for 24 h with ESVR (52 *μ*g/mL) diluted in RPMI 1640 solution with 0.1% DMSO, and RPMI 1640 solution with 0.1% DMSO was used as a control. Subsequently, the cells were trypsinized and incubated with 10 *μ*M H_2_DCFDA for 30 min at room temperature and protected from light. After the incubation period, the fluorescence, related to the ROS levels, was acquired in 15,000 events in the Accuri C6 flow cytometer (Becton Dickinson, San Jose, CA) and analyzed using the software FlowJo v8.7. (Ashland, US).

### 2.11. Pan-Caspase, Intracellular Calcium, and Necrostatin-1 Inhibition

To assess the involvement of caspases, intracellular calcium, and RIPK1 in ESVR-promoted cell death, B16F10-Nex2 cells (5 × 10^3^ cells/well) were pretreated for 1 h with carbobenzoxy-valyl-alanyl-aspartyl-(O-methyl)-fluoromethylketone (Z-VAD-FMK; irreversible, cell-permeant pan-caspase inhibitor), 1,2-bis(2-aminophenoxy)ethane-N,N,N′,N′-tetraacetic acid tetra(acetoxymethyl ester) (BAPTA-AM; cell-permeant calcium chelator), or necrostatin-1 (NEC-1) inhibitor. Then, the cells were treated with ESVR (52 *μ*g/mL) diluted in RPMI 1640 solution with 0.1% DMSO for 24 h. RPMI 1640 solution with 0.1% DMSO was used as a control. After this period, the cell viability was determined using the MTT assay, which was previously described in Section 2.4.

### 2.12. Cell Cycle Phases

The distribution of cell cycle phases was assessed using the method described by Paredes-Gamero et al. in 2012 [28]. For this purpose, B16F10-Nex2 cells (6 × 10^4^ cells/well) were treated with ESVR (1/2 IC_50_ = 26 *μ*g/mL and IC_50_ = 52 *μ*g/mL) diluted in RPMI 1640 solution with 0.1% DMSO for 24 h. RPMI 1640 solution with 0.1% DMSO was used as a control. Subsequently, the cells were fixed and permeabilized as described above and incubated with RNAse (4 mg/mL) (Sigma-Aldrich, Germany) for 1 h at 37°C. For DNA labeling, the cells were incubated with SYTOX Green (5 *μ*g/mL) (Molecular Probes Inc., Oregon). The percentage of cells at each cell cycle phase (sub-G_0/_G_1_, S, and G_2_/M) was determined in 40,000 events in an Accuri C6 flow cytometer (Becton Dickinson, San Jose, CA). The results were analyzed using the software FlowJo v.8.7.

### 2.13. Animals

Male C57Bl/6 mice 4-6 weeks of age were obtained from the Centro de Desenvolvimento de Modelos Experimentais para Medicina e Biologia (Center for the Development of Experimental Models for Medicine and Biology-Federal University of São Paulo (CEDEME-UNIFESP), São Paulo, Brazil). In all experiments, the “Principles of Laboratory Animal Care” guidelines were followed (National Institute of Health (NIH) publication Number 85-23, revised in 1985), and animal experimentation was performed using protocols approved by the Animal Ethics Committee of the Federal University of São Paulo (UNIFESP) under number 1234/11.

### 2.14. *In Vivo* Antitumor Assay

Previously cultured B16F10-Nex2 melanoma cells (5 × 10^4^ cells/animals) were subcutaneously implanted in the lumbosacral region of C57Bl/6 mice (seven animals per group). From the second day of implantation, the mice were intraperitoneally injected with ESVR (520 *μ*g/mL) every other day for 30 days. The dose chosen was 10 times higher than the IC_50_ observed in the *in vitro* assays. The mice from the control group were intraperitoneally injected with the vehicle RPMI 1640 with 0.1% DMSO. The tumor volume was monitored after the 16th day of treatment, and the tumor diameter was measured three times a week. The tumor volume was determined using the following formula:

(2) $$\begin{array}{c} \text{Tumor volume }\left({\text{mm}}^{3}\right)=\text{larger diameter }\times \text{ smaller }{\text{diameter}}^{2}\times 0.52. \end{array}$$

### 2.15. *In Vivo* Pulmonary Metastasis Evaluation

The experiment was conducted according to Pereira et al. in 2016 [30] with minor modifications. Thus, 5 × 10^5^ B16F10-Nex2 melanoma cells were injected through the caudal vein into C57Bl/6 mice (five animals per group). From the second day of implantation, the mice were intraperitoneally injected with ESVR (520 *μ*g/mL) every other day for 14 days. The mice from the control group were intraperitoneally injected with vehicle RPMI 1640 with 0.1% DMSO. On the 15th day, the mice were anesthetized and euthanized. The lungs were removed, and the lung nodules were counted using a stereoscope (Nikon SMZ745T), with the images recorded using a Ds-Fi2 camera.

### 2.16. Statistical Analysis

All data are expressed as the mean ± standard error of the mean (SEM). The half-maximal inhibitory concentrations (IC_50_) with confidence limits of 95% were determined by nonlinear regression using the software GraphPad Prism 6 software (San Diego, CA, USA). Significant differences between groups were determined using the unpaired Student's *t*-test (in apoptotic B16F10-Nex2 cell count, caspase-3 activity, determination of ROS levels, cell cycle phase, *in vivo* antitumor assay, and *in vivo* pulmonary metastasis evaluation) for comparison between two groups and analysis of variance (ANOVA) followed by Dunnett's test for comparison of two or more groups (in pan-caspase, intracellular calcium, and necrostatin-1 inhibition) using the GraphPad Prism 5 software (San Diego, CA, USA). The results were considered significant when *p* < 0.05.

## 3. Results

### 3.1. Phytochemical Composition of ESVR

**Figure 1:**
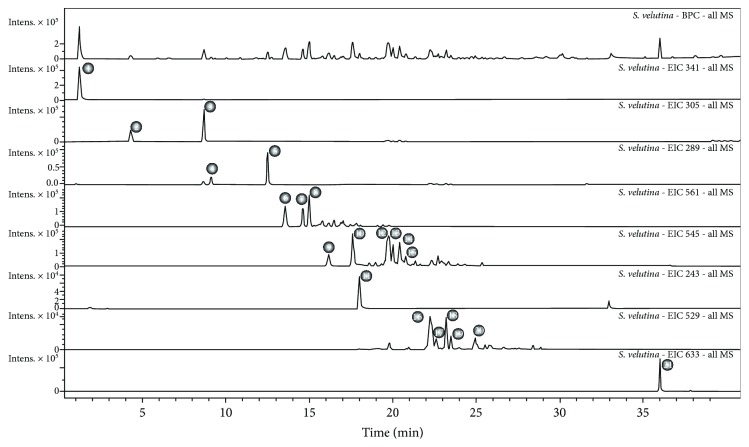
Characterization of the compounds identified in ESVR by UFLC-MS. Chromatograms with the base peaks and peaks identified in the extract.

Compounds relative to the twenty one chromatographic peaks were detected in the ethanolic extract of *S. velutina* roots, including sugar derivatives, gallocatechin, epigallocatechin, catechin, epicatechin, butiniflavan-(epi)gallocatechin, butiniflavan-(epi)catechin, piceatannol, cassiaflavan-(epi)gallocatechin, cassiaflavan-(epi) catechin, and dimeric tetrahydroanthracene derivatives (Figure 1 and Table 1).

### 3.2. Cell Viability Assay

**Figure 2:**
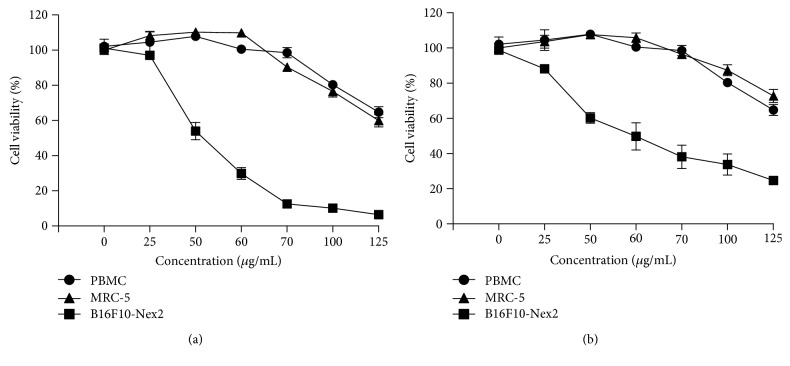
The cytotoxic effect of ESVR on PBMC, MRC-5, and B16F10-Nex2 cells treated with different ESVR concentrations for (a) 24 and (b) 48 h. The data are expressed as the means ± SEM in three independent experiments in triplicate.

Figure 2 shows that B16F10-Nex2 cells were sensitive to ESVR in a concentration-dependent manner after 24 h (IC_50_ of 52 *μ*g/mL) and 48 h (IC_50_ of 59 *μ*g/mL) of treatment. In both treatment periods, the ESVR decreased the number of viable cells. On the other hand, ESVR cytotoxicity was lower in the PBMC and MRC-5 cell lines than in B16F10-Nex2 cells; it was observed that even after 48 h of incubation with the highest dose tested (125 *μ*g/mL), the extract was cytotoxic to only 20-30% of the cells. Additionally, the effect of ESVR against human melanoma cell lines SK-Mel-28 (IC_50_ of 420.21 *μ*g/mL in 24 h and 330.48 *μ*g/mL in 48 h) and SK-Mel-103 (IC_50_ of 245.23 *μ*g/L in 24 h and 94.09 *μ*g/mL in 48 h) was evaluated (reported in supplementary Figure 1). However, in both SK-Mel cell lines the results were lower than those observed against B16F10-Nex2 cells. B16F10-Nex2 cells were chosen for the next analyses, since they were more sensitive to the action of ESVR.

### 3.3. Effects of ESVR on B16F10-Nex2 Cells

**Figure 3:**
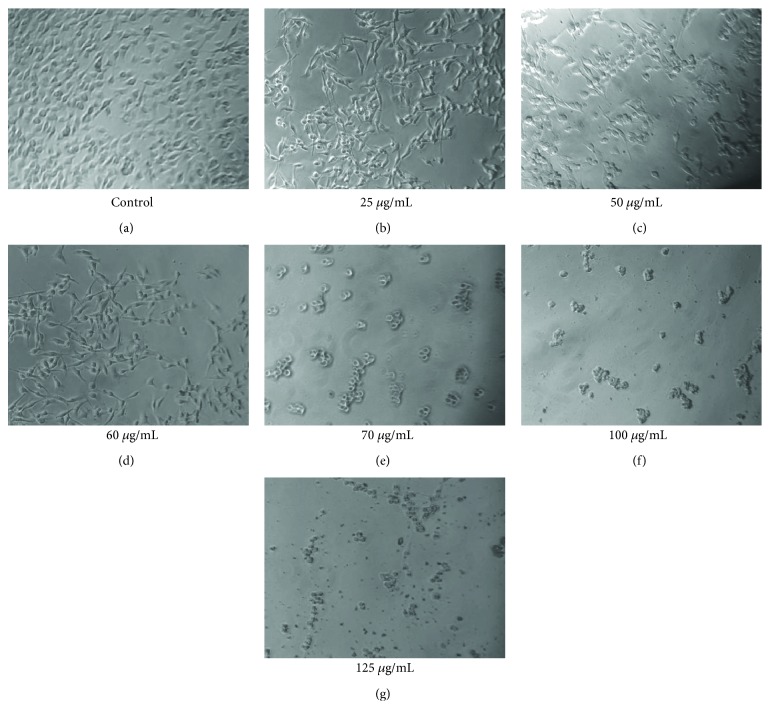
Reduced viability of B16F10-Nex2 cells treated with different ESVR concentrations for 24 h. Images are representative of those seen from at least three such fields of view per sample and three independent replicates.

Figure 3 shows the effects of different ESVR concentrations on B16F10-Nex2 cell viability and morphology after 24 h of treatment. At images of the 25 to 60 *μ*g/mL of the extract concentration, a reduction in the cell number was observed without changing the morphology of unaffected cells that remain attached to the extracellular matrix present at the well. On the other hand, cells treated with doses equal or higher than 70 *μ*g/mL of the extract showed a marked reduction in the cell number and strongly modified morphology of the remaining cells with loose attachment to the extracellular matrix.

### 3.4. Cell Death Profile

Cells were incubated with ESVR diluted in culture media at IC_50_ without annexin V-FITC or propidium iodide. However, the fluorescence of the extract was similar to that of the markers, thereby precluding the correct interpretation of these tests (data not shown).

### 3.5. Apoptotic B16F10-Nex2 Cell Count

**Figure 4:**
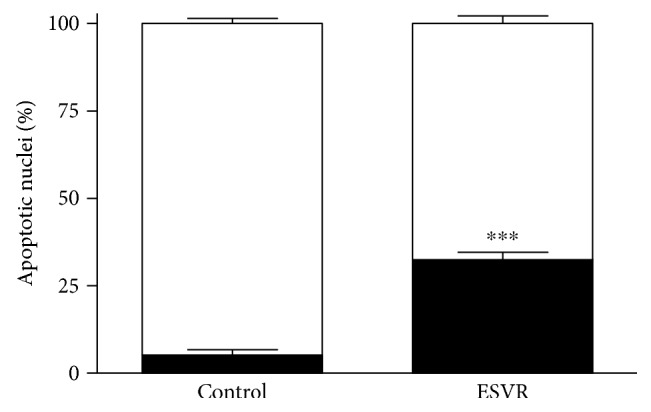
Count of apoptotic nuclei in B16F10-Nex2 cells treated for 24 h with 52 *μ*g/mL of ESVR. The data are expressed as the means ± SEM of four independent experiments in duplicate. ^∗∗∗^ *p* < 0.001 compared with control cells.

Nuclear morphological changes are characteristic of apoptotic cell death and can be determined by microscopy using specific fluorescence markers. In these analyses, the number of apoptotic B16F10-Nex2 cells treated with ESVR (52 *μ*g/mL) counted under confocal microscopy shows that the extract promoted nuclear damage in 32.4% of cells, whereas only 5.2% of untreated cells were damaged (Figure 4). Only nuclei that showed extreme chromatin condensation, DNA fragmentation, and high fluorescence intensity were considered apoptotic nuclei.

### 3.6. DNA Fragmentation

**Figure 5:**
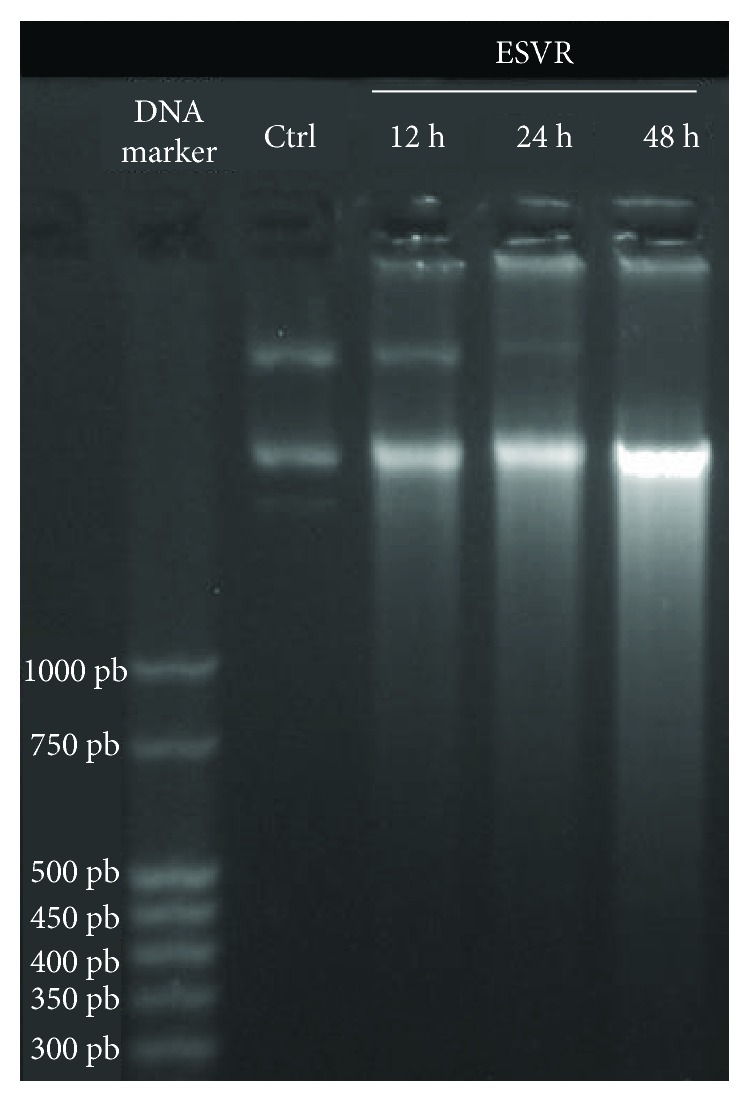
DNA fragmentation in B16F10-Nex2 cells analyzed by agarose gel electrophoresis after 12 h, 24 h, and 48 h of treatment with 52 μg/mL of ESVR. Ctrl = B16F10-Nex2 cells after 48 h without treatment with ESVR.

Compounds that activate cell death pathways such as apoptosis are able to induce DNA degradation. The DNA fragmentation data shown in Figure 5 demonstrate that ESVR-treated B16F10-Nex2 cells show time-dependent DNA fragmentation, which is observed after 12 h and is intensified after 24 h and 48 h of treatment. After these incubation periods, the control cells showed no sign of DNA fragmentation.

### 3.7. Caspase-3 Activity

**Figure 6:**
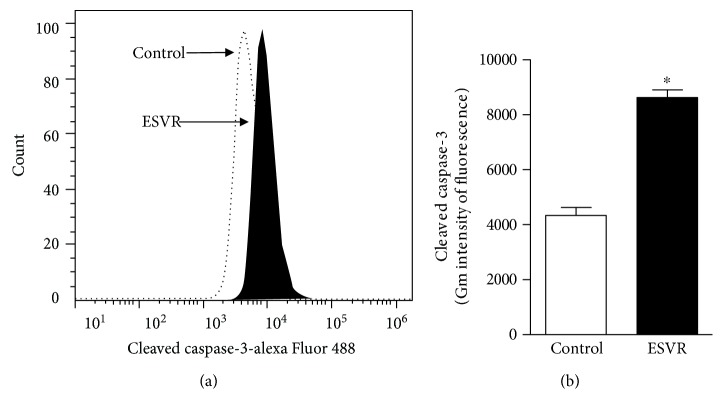
Caspase-3 activation by ESVR after 24 h represented in a histogram (a) and bar graph (b). The data are expressed as the means ± SEM of three independent experiments in duplicate. ^∗^ *p* < 0.05 compared with control cells.

Caspase-3 is an effector caspase that plays a central role in the execution phase of apoptosis. Caspase-3 activation was assessed to identify the possible cell death pathways activated by ESVR in melanoma cells. In this assay, Figure 6(a) shows right-shifted fluorescence values, thus confirming caspase-3 activation and indicating apoptosis-induced cell death. The increase in cleaved caspase-3 in ESVR-treated B16F10-Nex2 cells was twice as high as that in control cells (Figure 6(b)).

### 3.8. Determination of the Reactive Oxygen Species (ROS) Levels

**Figure 7:**
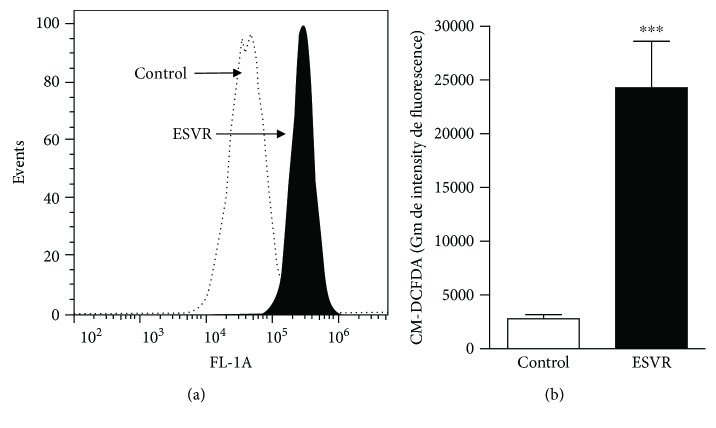
Determination of the levels of ROS in B16F10-Nex2 cells treated for 24 h with 52 *μ*g/mL of ESVR, represented in a histogram (a) and bar graph (b). The data are expressed as the means ± SEM of three independent experiments in duplicate. ^∗∗∗^ *p* < 0.001 compared with control cells.

ROS were evaluated in this study to verify whether they were involved in ESVR-induced cell death. The levels of ROS increased in ESVR-treated cells, as shown by the right-shifted fluorescence values (Figure 7(a)). The mean values obtained in the fluorescence intensity were 24.271 ± 4.309 for treated cells with ESVR and 2.787 ± 408 for the control cells (Figure 7(b)). The ROS levels increased 8.7-fold in B16F10-Nex2 cells treated with the extract after 24 h of incubation compared with control cells without treatment.

### 3.9. Pan-Caspase, Intracellular Calcium, and Necrostatin-1 Inhibition

**Figure 8:**
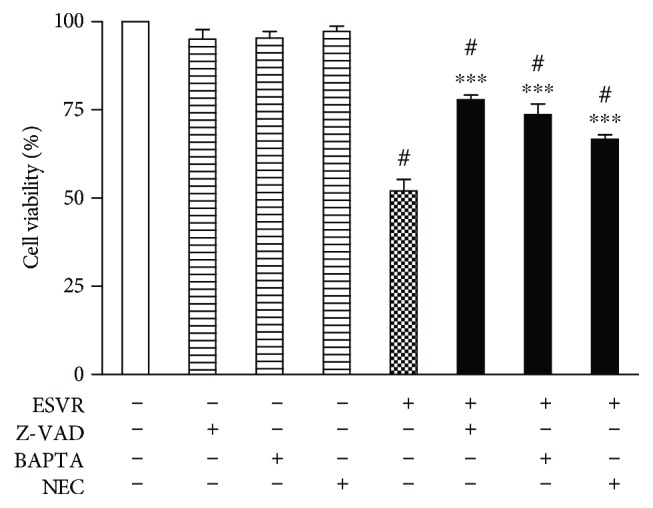
Effect of pan-caspase (Z-VAD-FMK), intracellular calcium channels (BAPTA-AM), and necrostatin-1 (NEC) inhibitors on B16F10-Nex2 cells treated or untreated for 24 h with ESVR at a concentration of 52 *μ*g/mL. The data are expressed as the means ± SEM of three independent experiments in duplicate. ^#^ *p* < 0.05 compared with negative control cells and ^∗∗∗^ *p* < 0.001 compared with ESVR-treated cells.

Aiming to identify cell death modalities induced by ESVR in B16F10-Nex2 cells, different markers of apoptosis and necrosis were analyzed.  Figure 8 shows that neither control B16F10-Nex2 cells nor the B16F10-Nex2 cells treated with only the inhibitors showed changes in cell viability. Conversely, cells treated with ESVR (52 *μ*g/mL) for 24 h showed 52.0 ± 3.3% viable cells. This result was partially reversed in the presence of the inhibitors Z-VAD-FMS (77.9 ± 1.4%), BAPTA-AM (73.7 ± 3.1%), and NEC-1 (66.7 ± 1.2%).

### 3.10. Cell Cycle Phases

**Figure 9:**
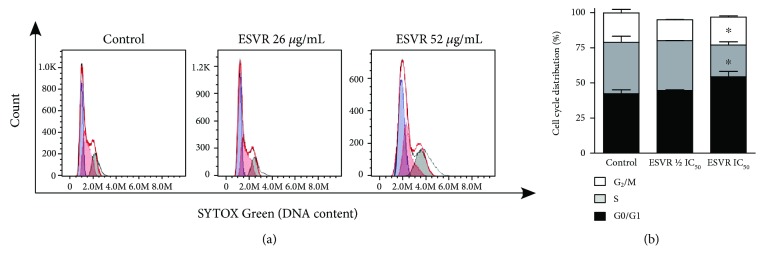
Histograms (a) and a bar graph (b) representative of the cell cycle distribution of control (untreated) B16F10-Nex2 cells and B16F10-Nex2 cells treated for 24 h with 1/2 IC_50_ = 26 *μ*g/mL and IC_50_ = 52 *μ*g/mL ESVR. The data are expressed as the means ± SEM of four independent experiments. ^∗^ *p* < 0.05 compared with control cells.

Cell cycle control in tumor cells is considered an important therapeutic target for the treatment of cancer. Thus, demonstrating the effects of the extract on the progression of the cell cycle will contribute to a better understanding of its mechanisms of action.  Figure 9(a) shows histograms of the cell cycle distribution of control B16F10-Nex2 cells and B16F10-Nex2 cells treated with 1/2 IC_50_ = 26 *μ*g/mL and IC_50_ = 52 *μ*g/mL ESVR for 24 h. The control cells and cells treated with 26 *μ*g/mL of ESVR showed no differences in cell cycle distribution (Figure 9(b)). The comparison between cells treated with 52 *μ*g/mL of ESVR and control cells shows that ESVR promoted cell cycle arrest at the G_0_/G_1_ phase (54.3 ± 3.8% versus 42.3 ± 2.6%, ^∗^ *p* < 0.05) and decreased the percentage of S phase cells (22.5 ± 2.2% versus 36.6 ± 4.2%, ^∗^ *p* < 0.05) without changing the number of cells in the G_2_/M phase (19.9 ± 0.8% versus 20.9 ± 2.4%) (Figure 9(b)).

### 3.11. *In Vivo* Effect of ESVR on the Tumor Volume

**Figure 10:**
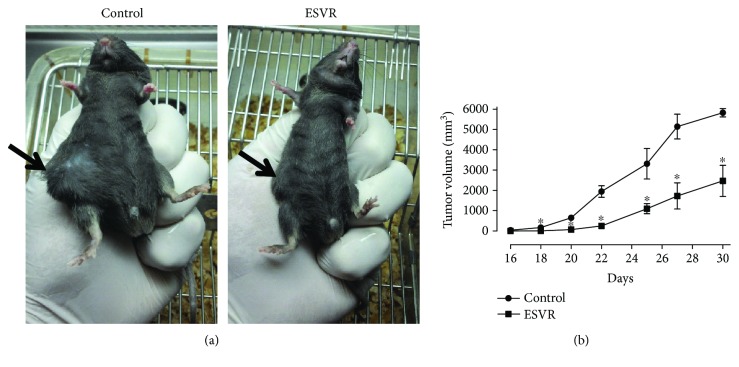
The effect of ESVR on the tumor volume of B16F10-Nex2 cells induced in C57Bl-6 mice. Representative images of 30-day tumors (arrows) in (a) control animals treated with RPMI 1640 medium and animals treated with ESVR. (b) A representative plot of tumor volume progression during 30 days of treatment. The data are expressed as the means ± SEM (*n* = 7). ^∗^ *p* < 0.05 compared with the control group.

After observing that ESVR had a cytotoxic effect on B16F10-Nex2 cells *in vitro*, we next evaluated the effect of the extract *in vivo* during tumor progression. ESVR treatment of B16F10-Nex2-inoculated mice significantly delayed subcutaneous tumor development in all animals analyzed (Figure 10(a)).  Figure 10(b) shows that the mean tumor volume of mice 30 days after the treatment was 57.5% smaller than the tumor volume of the control mice.

### 3.12. *In Vivo* Effect of ESVR on Pulmonary Metastasis

**Figure 11:**
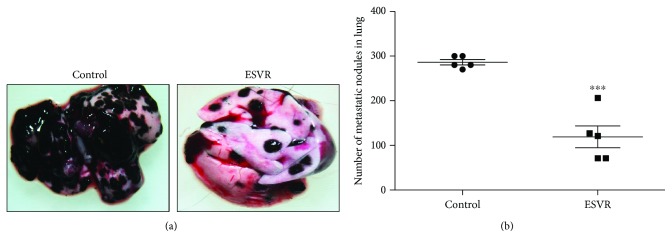
The effect of ESVR on the pulmonary metastasis of B16F10-Nex2 cells induced in C57Bl/6 mice. Representative lung images of (a) control animals treated with RPMI 1640 and animals treated with ESVR. (b) A graph representing the number of pulmonary metastasis in all animals after 14 days of endogenous cell inoculation. The data are expressed as the means ± SEM (*n* = 5). ^∗∗∗^ *p* < 0.001 compared with the control group.

Next, we analyzed the effect of ESVR on metastatic B16F10-Nex2 cells developing in the lungs after endovenous inoculation at the caudal vein. It was observed that ESVR-treated animals showed 119 ± 25 pulmonary melanotic nodules 14 days after cell inoculation, while the control group showed 286 ± 6 pulmonary nodules, a 54% reduction (Figure 11).

## 4. Discussion

The search for new anticancer drugs with greater selectivity and lower adverse effects is an ongoing process. Natural compounds are among the alternatives that stand out as promising sources of new molecules with pharmacological potential. Accordingly, several anticancer drugs of natural origin are available on the market [31]. In this context, scientific studies have shown that Brazilian biodiversity due to its various biomes provides various natural compounds with anticancer potential both *in vitro* [32, 33] and *in vivo* [30, 34]. In the present study, we assessed the anticancer effects of the ethanolic extract of the roots of *S. velutina*, a plant species native to Brazil whose phytochemical composition and potential pharmacological applications have been poorly studied.

Phytochemical analysis of ESVR identified its main compounds as flavonoid derivatives of the catechin and piceatannol (active metabolite of resveratrol) groups as well as dimeric tetrahydroanthracene derivatives. These phenolic compounds derived from plant secondary metabolism exhibit great structural diversity and are responsible for innumerable biological activities, including anticancer properties [35, 36].

The assessment of the effect of ESVR on B16F10-Nex2 melanoma cell viability revealed a dose-dependent death profile. This effect was confirmed by microscopy, as shown by the activity of the extract at different concentrations. In addition, ESVR showed higher selectivity against B16F10-Nex2 cells than against human leukocytes (PBMC) or human fibroblasts (MRC5). This result is highly relevant because systemic collateral effects from chemotherapeutic agent activity are a consequence of reduced selectivity against tumor cells.

The ability of the extract to promote the death of melanoma cells may be related to the isolated or synergistic effects of its chemical constituents, since the main constituents and chemical classes identified in the ESVR are well described in the literature for their antitumor activities. Catechins are described by the ability to reduce the viability of breast carcinoma cells [37] and to promote the cytotoxic effect in B16F10 murine melanoma cells [38]. Anthraquinone compounds have been reported as promising therapeutic agents for the treatment of malignant melanoma for presenting high cytotoxicity against different malignant melanoma cells and low toxicity to melanocytes and other primary cell [39]. Piceatannol, defined as a promising therapeutic agent for the treatment of various cancers, inhibited growth and induced apoptosis in human melanoma cell lines [40].

Different studies report that catechins [41, 42], anthraquinones [39], and piceatannol [43, 44] induce apoptosis in tumor cells. Corroborating this is the analysis of the mechanism whereby ESVR-promoted B16F10-Nex2 cell death showed an increased number of apoptotic nuclei, which are characterized by chromatin condensation and DNA fragmentation, characteristic stages of death by apoptosis [45]. Apoptosis is considered a cell death process essential to homeostasis, mainly activated by extrinsic and intrinsic pathways [46]. In the extrinsic pathway, apoptotic receptors promote extracellular signaling. Conversely, in the intrinsic pathway, activation occurs in response to intracellular damage mediated by the mitochondria [47], a process characterized by the release of proapoptotic proteins into the cytosol, thereby promoting caspase activation and nuclear apoptosis.

Caspases are essential apoptotic cell death mediators [48]. Among these proteases, caspase-3 is one of the main effectors of programmed cell death because it is directly involved in nuclear apoptosis and cell death [49]. In this study, ESVR-treated B16F10-Nex2 cells showed doubled activated caspase-3 levels. In addition, the pan-caspase inhibitor Z-VAD-FMK reduced the percentage of cell death, thus demonstrating the involvement of caspases in the mechanism of cell death promoted by the extract.

Assessment of the activity of inhibitors showed that calcium and the necroptosis pathway are among the mechanisms involved in ESVR-induced cell death. High cytoplasmic Ca^2+^ levels are responsible for mitochondrial membrane permeabilization with cytochrome c release, thereby enhancing the signs of apoptosis [50–52].

Necroptosis is a form of cell death that shows characteristics of necrosis, but unlike necrosis, it may be regulated by receptor-interacting proteins 1 (RIP1) and 3 (RIP3) [53]. Furthermore, recent studies show that oxidative stress may promote necroptosis activation [54, 55]. Although necroptosis is not the main mechanism of death characterized by the action of ESVR, this finding is interesting, one which can be an alternative form of cell death to populations of cells exhibiting resistance to death by apoptosis.

Conversely, cancer cells also have a persistent prooxidative state and high ROS levels [56]. This different metabolism promotes an adaptive response that plays a key role in cancer cell proliferation, cell death signaling disruption, metastasis, and resistance to antitumor drugs [57, 58]. Nevertheless, cancer cells become vulnerable to prooxidant agents that further increase ROS levels, thus promoting cell death [56].

Some flavonoids, the main compounds of ESVR, are described in the literature as prooxidant agents in cancer cells [41, 59] because they can directly increase ROS production, resulting in superoxide radical formation [60], a mechanism whereby ESVR may have contributed to B16F10-Nex2 cell death because the intracellular ROS levels were high. Furthermore, anthracene derivatives, another group of compounds identified in ESVR, are described for inducing apoptosis by increasing ROS production [54].

Another activity promoted by the extract was cell cycle arrest at the sub-G_0_/G_1_ phase, accompanied by a decrease in the percentage of S phase cells. Some flavonoids, such as catechins, can cause cell cycle arrest at the G_0_/G_1_ phase of the cell cycle [55, 61]. Piceatannol decreases cyclin-dependent kinase 1 (CDK1), which is responsible for cell cycle progression from the G_1_ phase to the S phase [62]. Thus, considering the importance of the cyclin-dependent kinases involved in cell cycle regulation and the uncontrolled cell proliferation in tumor cells, compounds capable of inhibiting the cell cycle progression of these cells may be important alternatives for tumor volume control [63, 64].

In this study, after confirmed *in vitro* antitumor action in B16F10-Nex2 murine melanoma cells, we demonstrated that ESVR produces antitumor activity on tumor volume progression and pulmonary nodule formation *in vivo*. Our data from *in vivo* antitumor assessment showed that primary tumor progression in ESVR-treated mice decreased by more than 50% compared with control mice. Cutaneous melanoma is one of the most aggressive forms of cancer, and no fully effective pharmacological therapy for advanced-stage metastatic melanoma is currently available [65, 66]. In these cases, metastases are responsible for the poor prognosis [67], affecting several organs, such as the bones, liver, and lungs [68]. The progression of pulmonary metastasis in ESVR-treated mice was markedly reduced, as observed in the formation of pulmonary nodules. This result may be related to the ability of the extract to reduce the migration and invasion of melanoma cells into the lungs. These effects may be related to the chemical constituents of ESVR, since epigallocatechin (one of the identified catechins), anthraquinones, and resveratrol (precursor molecule of piacetannol) are compounds reported to inhibit tumor growth [69–71] and progression of pulmonary metastasis [70] in animal models for melanoma. In addition, phenolic compounds are well described because they hamper metastasis by decreasing metalloprotease-9 expression in murine melanoma cells [72, 73]. Moreover, other studies showed that epigallocatechin can inhibit genes that synthesize proteins related to extracellular matrix degradation and cellular mobility, thereby reducing the process of melanoma metastasis [74].

In conclusion, this study demonstrated that ESVR contains flavonoid derivatives of catechins, anthraquinones, and piceatannol among its chemical constituents and promotes B16F10-Nex2 melanoma cell death via apoptosis induced by caspase-3 activation, the elevation of intracellular calcium and ROS levels, and cell cycle arrest at the G_0_/G_1_ phase. Furthermore, the extract showed *in vivo* antitumor activity in models of primary tumor volume progression and pulmonary nodule formation. These promising results open the door for further studies, both with the crude extract and with fractions isolated from *Senna velutina* roots, exploring its potential use in the treatment of melanoma and other cancers.

## Figures and Tables

**Table 1:** ESVR chemical profile as analyzed by UFLC-MS (negative mode).

Peak	Retention time	UV	Molecular formula	(M-H)	PPM error	MS/MS	Compound
1	1.1	—	C_12_H_20_O_11_	341.1086	0.6	341: 179	Sugar derivative
2	4.2	270	C_15_H_14_O_7_	305.0657	3.2	305: 261, 221, 219, 179, 167, 165	Gallocatechin
3	8.6	270	C_15_H_14_O_8_	305.0660	2.3	305: 261, 221, 219, 179, 167, 165	Epigallocatechin
4	9.1	280	C_15_H_14_O_9_	289.0709	3.0	289: 245, 205, 203	Catechin
5	12.5	280	C_15_H_14_O_10_	289.0714	1.3	289: 245, 205, 203	Epicatechin
6, 7, 8	12.5/13.5/14.6	278	C_30_H_26_O_11_	561.1402/561.1402	1.1	561: 407, 305, 177, 165	Butiniflavan-(epi)gallocatechin
9, 10	16.1/17.6	280	C_30_H_26_O_10_	545.1453/545.1445	0.7	545: 391, 289, 245	Butiniflavan-(epi)catechin
11	18	289/321	C_14_H_12_O_4_	243.0662	0.3	243: 201, 159	Piceatannol
12, 13, 14, 15	19.6/20/20.4/20.7	280	C_30_H_26_O_10_	545.1441	2.3	545: 305, 239, 165	Cassiaflavan-(epi)gallocatechin
16, 17, 18, 19, 20	22.2/22.7/23.2/23.5/24.9	280	C_30_H_26_O_9_	529.1488	3.0	529: 289, 245, 239, 203	Cassiaflavan-(epi)catechin
21	36.1	279/320/406	C_34_H_34_O_12_	633.1992	2.2	633: 615, 597, 579, 557, 555, 539, 317, 299, 298, 259	Dimeric tetrahydroanthracene derivative

## Data Availability

The experimental data used to support the findings of this study are included within the article.

## Supplementary Materials

Supplementary Materials Figure S1: the cytotoxic effect of ESVR on human melanoma cell lines SK-Mel-28 and SK-Mel-103 treated with different concentrations for (A) 24 h and (B) 48 h. The data are expressed as the means ± SEM in three independent experiments in triplicate.
