# Supplementary material for: Ethanolic Extract of Senna velutina Roots: Chemical Composition, In Vitro and In Vivo Antitumor Effects, and B16F10-Nex2 Melanoma Cell Death Mechanisms
Source: Oxid Med Cell Longev. 2019 Jun 12;2019:5719483. doi: 10.1155/2019/5719483 (PMC6594258; doi:10.1155/2019/5719483)
Supplement: Supplementary Materials — Figure S1: the cytotoxic effect of ESVR on human melanoma cell lines SK-Mel-28 and SK-Mel-103 treated with different concentrations for (A) 24 h and (B) 48 h. The data are expressed as the means ± SEM in three independent experiments in triplicate. [file 5719483.f1.docx]

**Supplementary Figure S1.** The cytotoxic effect of ESVR on human melanoma cell lines SK-Mel-28 and SK-Mel-103 cells treated with different concentrations for (A) 24h and (B) 48h. The data are expressed as the means ± SEM in three independent experiments in triplicate.
